# Calcium to magnesium intake ratio and non-alcoholic fatty liver disease development: a case-control study

**DOI:** 10.1186/s12902-021-00721-w

**Published:** 2021-03-18

**Authors:** Hadi Emamat, Hamid Ghalandari, Ali Saneei Totmaj, Hadith Tangestani, Azita Hekmatdoost

**Affiliations:** 1 Student Research Committee, Department of Clinical Nutrition and Dietetics, Faculty of Nutrition Sciences and Food Technology, National Nutrition and Food Technology Research Institute, Shahid Beheshti University of Medical Sciences, Tehran, Iran; 2 Department of Clinical Nutrition and Dietetics, Faculty of Nutrition Sciences and Food Technology, National Nutrition and Food Technology Research Institute, ShahidBeheshti University of Medical Sciences, 19395 − 4741, No 7, West Arghavan St., Farahzadi Blvd, 1981619573 Tehran, Iran; 3 Department of nutrition, School of Public Health, Iran University of Medical Sciences, Tehran, Iran; 4 Department of Nutrition, Persian Gulf Tropical Medicine Research Center, Bushehr University of Medical Sciences, Bushehr, Iran

## Abstract

**Background:**

Non-alcoholic fatty liver disease (NAFLD) is the most common chronic liver disease worldwide. Adoption of sedentary life style and westernized diet are shown to be associated with development of NAFLD. Since previous studies suggested that calcium (Ca) to magnesium (Mg) ratio intake is associated with some chronic diseases including dyslipidemia and insulin resistance, we designed this study to find any possible association between this ratio and NAFLD development.

**Methods:**

The NAFLD was diagnosed using Fibroscan according to a CAP cut-off value of 263 dB/m. Dietary intakes of one hundred and ninety-six patients with incident NAFLD diagnosis, and eight hundred and three controls without NAFLD were assessed using a valid food frequency questionnaire (FFQ). Dietary nutrients were calculated using Nutritionist IV software.

**Results:**

Age of the study population (57 % female) was 43.2 ± 14.1 years. In addition, energy-adjusted daily calcium to magnesium intake ratio was 2.34 ± 0.57 and 2.73 ± 0.69 for control and case groups, respectively. In the multivariable-adjusted model, after adjustment for potential confounding variables; including, age, gender, BMI, alcohol consumption, smoking, diabetes, physical activity, energy, dietary fiber, carbohydrate, fat, and protein intakes, participants in the third (Q3) and fourth (Q4) quartile of Ca/Mg ratio intake had a greater development of incidental NAFLD compared to the lowest quartile (Q1) [(OR = 2.86; 95 % CI: 1.20–6.81), (*P*-value = 0.017) and (OR = 5.97; 95 % CI: 2.54–14.01), (*P*-value < 0.001) for Q3 and Q4 compared to the Q1, respectively]. Moreover, energy-adjusted Ca to Mg intake ratio was positively correlated with plasma level of ALT (*r* = 0.18; *P* = 0.01); contrarily, it had no correlation with plasma levels of AST.

**Conclusions:**

The current study revealed that higher dietary Ca to Mg intake ratio is associated with a greater development of NAFLD. Further interventional studies are needed to confirm the causal relationship of the Ca/Mg ratio intake and development of NAFLD.

## Background

Non-alcoholic fatty liver disease (NAFLD) includes the entire spectrum of fatty liver disease in patients without significant alcohol consumption, ranging from fatty liver to steatohepatitis to cirrhosis [1]. NAFLD is one of the most common causes of liver cirrhosis and liver cancer [2–4]. The mortality rate of cirrhosis and cirrhosis-related diseases such as liver cancer has increased over the past 35 years worldwide [5]. Obesity, type 2 diabetes, overmedication, and exposure to the toxic substances constitute the etiology of NAFLD [6–8]. Patients with metabolic syndrome are much more likely to develop NAFLD [9, 10].It has been estimated that NAFLD will be one of the leading causes of liver disease and its consequent mortality by 2030, along with the obesity epidemic [11]. There is also a population so called “lean NAFLD” [12]. It has been shown that weight reduction is also beneficial in this population [13]. The first line in the treatment of NAFLD is dietary interventions and reducing central obesity [14]. Although the pathogenic role of macronutrients in NAFLD and obesity are clear, the contribution of micronutrients in the pathogenesis of NAFLD has garnered less attention than that of obesity [15]. Overall, micronutrients play an important role in NAFLD pathogenesis [16].

Magnesium (Mg) is an abundant cation in the human body that plays essential roles in multiple physiological pathways such as cellular energy metabolism, inflammation, nucleic acid metabolism, protein synthesis, and electrolyte balance [17]. Serum Mg concentration is strictly controlled; however, hypomagnesemia may occur as a result of increased renal excretion or decreased digestive absorption of magnesium [18]. Hypomagnesemia and subclinical Mg deficiency are associated with osteoporosis, seizure, depression, diabetes mellitus, hypertension, dyslipidemia, and colorectal cancer [19–21]. On the other hand, an observational study found that higher intakes of Mg are associated with a lower development of NAFLD in young adults [22].

Calcium (Ca) is another major mineral that is mainly deposited in bone and plays a key role in various biological processes [23]. Ca and Mg could be regarded as antagonist to each other in various biochemical pathways [24]. Studies show that altered serum calcium is associated with dyslipidemia and insulin resistance [25]. Previous studies on human subjects indicate that a high Ca intake may affect the absorption rate of Mg, which in turn, can be associated with an increased risk of several diseases [26–29]. Taking all these facts into account, it is plausible to consider the intake ratio of calcium to magnesium as a potential contributor to NAFLD pathogenesis [28].

Due to the novelty of the idea, we decided to examine the possible relationship between calcium to magnesium intake ratio and development of NAFLD in this study.

## Materials/subjects and methods

This case–control study was conducted at two clinics in Tehran province of Iran. The study included 196 cases with NAFLD and 803 controls. These participants were selected with the convenience sampling procedure. We conducted this case-control study on patients with recently diagnosed NAFLD and age-matched controls subjects from the same clinic. The absence of hepatic steatosis in individuals in the control group was determined using the ultrasound exam. The presence of NAFLD in our cases was confirmed by a gastroenterologist; moreover, the inclusion criteria included having a Controlled Attenuation Parameter (CAP) score of more than 263, fibrosis score more than 7, determined by the Fibroscan device, and the intake of alcohol less than 20 gram/day in women, and less than 30 gram/day in men. Fibroscan is an ultrasound device calibrated to measure hepatic steatosis levels producing an index called CAP; CAP score indicates the level of infiltration of fat in hepatocytes and has sensitivity and specificity ranged between 78 and 100 % [30]. All tests were performed by one operator with Fibroscan (EchoSens, Paris, France) device. Patients fasted for at least 3 h before the test. Patients were lying on the bed for at least 3 min before the test. The probes were calibrated before starting work. The other secondary causes of hepatic fat accumulation such as long-term use of a steatogenic medication (e.g., mipomersen, lomitapide, amiodarone, methotrexate, tamoxifen, corticosteroids, valproate and anti-retroviral medicines), or Hepatitis C (genotype 3) or Wilson disease or lipodystrophy or starvation or abetalipoproteinemia or Reye syndrome or inborn errors of metabolism were our exclusion criteria [1].

A validated food frequency questionnaire (FFQ) was used to assess the dietary intake of the participants [31]. Additionally, calorie-density of the two minerals (the amount of each mineral in an energy intake of 1000 Kcals) and their ratio were calculated for further analysis.

Baseline characteristics of all participants, including data on age, employment, marital status, education, smoking, past medical history, current use of medications and other factors were obtained by the interviewers. Physical activity level was assessed using physical activity questionnaire and was later quantified as metabolic equivalent hours per week (METs h/wk). Observational studies were reported according to STROBE guidelines. 

Baseline characteristics and dietary intakes between two study groups were compared using student t-test for continuous variables and chi-square for categorical variables.We used SPSS (Version 21.0; Chicago, IL, USA) software to conduct the satatistical analyses.The study participants were divided into four categories based onquartiles of daily energy-adjusted calcium to magnesium ratio and the lowest quartile was set as the reference category in order to evaluate the association between energy-adjusted daily calcium to magnesium intake ratio and NAFLD development. We took advantage of ANOVA test to compare the inter-quartile relationships. Odds ratios (ORs) and 95 % confidence intervals (CIs) were calculated using multiple logistic regression analysis. Analyses were adjusted for all known confounding factors. P-value less than 0.05 considered as significant.

## Results

Baseline participants’ characteristics in case and control group are shown in Table 1. The participants in case group had higher levels of BMI, fasting blood sugar (FBS), triglyceride (TG), low-density lipoprotein–cholesterol (LDL-c), physical activity (PA), alanine aminotransferase (ALT), aspartate aminotransferase (AST), smoking, diabetes, male, protein, calcium and calcium to magnesium intake ratio and lower levels of high-density lipoprotein–cholesterol (HDL-c), carbohydrate and fat intake (P < 0.05). Baseline characteristics regarding energy-adjusted daily calcium to magnesium intake ratio quartiles are presented in Table 2. Mean ± SD age of the study population (57 % female) was 43.2 ± 14.1 years. In addition, mean ± SD of energy-adjusted daily calcium to magnesium intake ratio was 2.34 ± 0.57 and 2.73 ± 0.69 for control and case groups, respectively.

**Table 1:** Baseline participants’ characteristics in case and control group

Characteristics	Case (*n* = 196)	Control (*n* = 803)	*P* value ^*^
Age (years)	42.3 ± 11.9	43.5 ± 14.5	0.214
Male (%)	51.5	41.0	0.007
Body mass index (kg/m^2^)	35.7 ± 10.6	27.7 ± 4.5	< 0.001
Fasting blood glucose (mg/dl)	108.5 ± 37.6	90.2 ± 29.4	< 0.001
Triglycerides (mg/dl)	176.0 ± 117.9	132.0 ± 81.8	< 0.001
Total cholesterol (mg/dl)	185.7 ± 52.8	177.5 ± 38.9	0.118
High Density Lipoprotein–Cholesterol (mg/dl)	41.9 ± 16.1	47.7 ± 10.5	0.001
Low Density Lipoprotein–Cholesterol(mg/dl)	120.9 ± 41.6	104.0 ± 31.9	< 0.001
Physical activity (MET(hour/week))	31.0 ± 3.2	34.2 ± 3.1	< 0.001
Alanine aminotransferase (U/L)	55.71 ± 8.27	20.49 ± 7.59	< 0.001
Aspartate aminotransferase (U/L)	33.86 ± 23.69	22.14 ± 7.72	< 0.001
Current alcohol usage (%)	12.8	9.1	0.07
Current smoking (%)	89.7	18.8	< 0.001
Diabetes (%)	16.6	6.8	< 0.001
Energy (Kcal/d)	2757 ± 961.1	2804 ± 840.7	0.499
Protein (% of energy)	15.8 ± 2.9	14.1 ± 2.3	< 0.001
Carbohydrate (% of energy)	58.2 ± 6.3	59.8 ± 13.3	0.014
Fat (% of energy)	29.2 ± 5.3	33.8 ± 5.7	< 0.001
Total dietary fiber (g/d)	46.29 ± 19.07	55.69 ± 46.11	0.08
Magnesium (mg/d)	484 ± 155	509 ± 173	0.09
Calcium (mg/d)	1389 ± 458	1114 ± 390	< 0.001
Calcium to Magnesium ratio	2.79 ± 0.68	2.34 ± 0.57	< 0.001

Quantitative data are presented as mean ± SD and qualitative data reported as percent

^*^Independent sample t-test and chi-square or Fisher exact test for quantitative and qualitative parameters, respectively

**Table 2:** Baseline participants’characteristics across quartiles of energy-adjusted daily calcium to magnesium ratio intake

	Quartiles of energy-adjusted daily calcium to magnesium ratio intake				
**Characteristics**	**Q1 (*n* = 250)**	**Q2 (*n* = 249)**	**Q3 (*n* = 250)**	**Q4 (*n* = 250)**	*P* value^*^
Age (years)	45.1 ± 13.7	41.9 ± 14.3	43.5 ± 14.1	43.3 ± 14.3	0.16
Male (%)	42.4	45	46.8	38	0.21
Body mass index (kg/m^2^)	28.7 ± 5.2	27.8 ± 5.3	28.4 ± 5.8	29.1 ± 6.7	0.006
Fasting blood glucose (mg/dl)	94.4 ± 34.6	92.9 ± 34.1	89.8 ± 30	92.3 ± 27.1	0.44
Triglycerides (mg/dl)	139 ± 92.1	140 ± 109.6	133 ± 71.9	131 ± 67.9	0.69
Total cholesterol (mg/dl)	179 ± 36.3	178 ± 44.4	175 ± 33.9	183 ± 37.1	0.33
High Density Lipoprotein–Cholesterol (mg/dl)	46.7 ± 10.2	46.4 ± 11.2	47.2 ± 10.3	49.1 ± 11.8	0.44
Low Density Lipoprotein–Cholesterol (mg/dl)	104 ± 30.7	103 ± 35.6	101 ± 29.6	107 ± 29.5	0.033
Physical activity (MET(hour/week))	32.1 ± 4.4	34.9 ± 3.3	31 ± 3.9	33.8 ± 2.1	0.22
Alanine aminotransferase (U/L)	22.9 ± 23.1	23.7 ± 28.6	24.2 ± 14.1	23.4 ± 19.1	0.08
Aspartate aminotransferase (U/L)	23.1 ± 10.3	23.6 ± 13.6	23.5 ± 9.2	22.1 ± 8.9	0.95
Current alcohol usage (%)	9.6	10.4	9.2	10	0.97
Current smoking (%)	25.6	32.1	28.8	44.2	< 0.001
Diabetes (%)	10.8	7.6	7.6	8.9	0.53
Energy (Kcal/d)	2712 ± 857	2846 ± 815	2876 ± 930	2758 ± 769	0.024
Protein (% of energy)	14.8 ± 1.5	14.4 ± 3.3	15.1 ± 1.7	14.9 ± 6.3	0.16
Carbohydrate (% of energy)	58.6 ± 12.3	56.8 ± 10.9	54.9 ± 8.2	59.1 ± 8.7	0.025
Fat (% of energy)	29.4 ± 1.3	32.5 ± 2.1	30.9 ± 7.6	29.1 ± 4.2	0.042
Total dietary fiber (g/d)	43.6 ± 16	45.4 ± 17.6	50.1 ± 36.8	50.1 ± 21.1	0.001
Magnesium (mg/d)	521 ± 178	508 ± 154	491 ± 167	434 ± 130	< 0.001
Calcium (mg/d)	879 ± 315	1106 ± 340	1249 ± 419	1404 ± 412	< 0.001
Calcium to Magnesium ratio	1.69 ± 0.20	2.17 ± 0.11	2.54 ± 0.12	3.25 ± 0.38	< 0.001

Quantitative data are presented as mean ± SD and qualitative data reported as percent

^*^one way ANOVA test and chi-square or Fisher exact test for quantitative and qualitative parameters, respectively

Inter-quartile analyses revealed that participants with higher daily calcium to magnesium intake ratio had significantly higher prevalence of smoking (*P* < 0.001), higher levels of BMI (*P* = 0.006), and higher levels of LDL-c (*P* = 0.033) compared to those with lower daily calcium to magnesium intake ratio. Significant differences were also detected regarding dietary intakes of energy (*P* = 0.024), carbohydrate (P < 0.025), fat (*P* = 0.042), and dietary fiber (*P* = 0.001) when inter-quartile data were analyzed. Compared by the same criteria, no other statistically significant differences were found.

Firstly, we evaluated the associations of energy adjusted calcium and magnesium intake with the development of NAFLD (Tables 3 and 4). Regarding to the energy adjusted calcium intake, in the final model adjustments for potential confounding variables; including, age, gender, BMI, alcohol consumption, smoking, diabetes, physical activity, energy, dietary fiber, carbohydrate, fat, and protein intakes, a positive association was detected between dietary calcium intake and the development of NAFLD in the third (Q3) and fourth (Q4) quartile compared to the lowest quartile (Q1) [(OR = 3.03; 95 % CI: 1.25–7.36), (*P*-value = 0.014) and (OR = 5.41; 95 % CI: 3.87–10.87), (*P*-value < 0.001) for Q3 and Q4 compared to the Q1, respectively] (Table 3). However, analyzes showed a negative association between the energy adjusted dietary magnesium intake and the development of NAFLD in fourth (Q4) quartile compared to the lowest quartile (Q1) [(OR = 0.89; 95 % CI: 1.25–4.78), (*P*-value = 0.043)] (Table 4).

**Table 3:** The development of non- alcoholic fatty liver disease across quartiles of energy-adjusted daily calcium intake

	Energy-adjusted daily calcium intake						
	**Q1(*n* = 249)**	**Q2(*n* = 250)**	*P*-value^*^	**Q3(*n* = 250)**	*P*-value^*^	**Q4(n = 250)**	*P*-value^*^
Cases/control	18/231	25/225		49/201		104/146	
Range of energy-adjusted Ca	135.8 to 347.3	347.7 to 407.3		407.4 to 473.4		473.9 to 1034.2	
^a^Model 1	1 (Ref)	1.42 (0.75–2.68)	0.27	3.12 (1.76–5.54)	**< 0.001**	9.14 (5.31–15.71)	**< 0.001**
^b^Model 2	1 (Ref)	1.43 (0.75–2.70)	0.28	3.34 (1.87–5.96)	**< 0.001**	9.96 (5.73–17.33)	**< 0.001**
^c^Model 3	1 (Ref)	1.92 (0.81–4.54)	0.13	2.81 (1.70–6.51)	**0.001**	6.34 (3.05–10.39)	**< 0.001**
^d^Model 4	1 (Ref)	1.99 (0.76–5.17)	0.15	3.03 (1.25–7.36)	**0.014**	5.41 (3.87–10.87)	**< 0.001**

Data are presented as odds ratio (95 %CI)

^*^Logistic regression

^a^crude model

^b^Adjusted for age and gender

^c^Additionally adjusted for body mass index, alcohol consumption, smoking, diabetes and physical activity

^d^Additionally adjusted for energy, dietary fiber, carbohydrate, fat, and protein intakes

The significance level: *P* < 0.05

**Table 4:** The development of non- alcoholic fatty liver disease across quartiles of energy-adjusted daily magnesium intake

	Energy-adjusted daily magnesium intake						
	**Q1(*n* = 250)**	**Q2(*n* = 249)**	*P*-value^*^	**Q3(*n* = 250)**	*P*-value^*^	**Q4(*n* = 250)**	***P*-value** ^*^
Cases/control	70/180	65/185		55/194		36/214	
Range of energy-adjusted Mg	86.31 to 154.40	154.43 to 174.70		174.71 to 196.63		196.65 to 329.96	
^a^Model 1	1 (Ref)	1.61 (0.57–2.34)	0.54	1.56 (0.78–3.49)	0.32	1.25 (0.61–5.56)	0.26
^b^Model 2	1 (Ref)	1.32 (0.75–3.32)	0.36	1.11 (0.65–4.31)	0.41	1.09 (0.74–6.32)	0.14
^c^Model 3	1 (Ref)	1.88 (0.53–4.53)	0.24	2.02 (0.48–6.21)	0.34	0.93 (0.81–5.70)	0.07
^d^Model 4	1 (Ref)	1.60 (0.43–2.34)	0.97	0.98 (0.71–3.61)	0.25	0.89 (1.25–4.78)	**0.043**

Data are presented as odds ratio (95 %CI)

^*^Logistic regression

^a^crude model

^b^Adjusted for age and gender

^c^Additionally adjusted for body mass index, alcohol consumption, smoking, diabetes and physical activity

^d^Additionally adjusted for energy, dietary fiber, carbohydrate, fat, and protein intakes

The significance level: *P* < 0.05

The data regarding the association between energy-adjusted calcium to magnesium intake ratio and the development of NAFLD is presented in Table 5. In the crude model, a positive association was detected between dietary calcium to magnesium intake ratio and the development of NAFLD in the third (Q3) and fourth (Q4) quartile compared to the lowest quartile (Q1) [(OR = 2.01; 95 % CI:1.21–3.34), (*P*-value = 0.007) and (OR = 4.33; 95 % CI:2.68–6.97), (*P*-value < 0.001) for Q3 and Q4compared to the Q1, respectively].In the multivariable-adjusted model, after adjustment for potential confounding variables; including, age, gender, BMI, alcohol consumption, smoking, diabetes, physical activity, energy, dietary fiber, carbohydrate, fat, and protein intakes, participants in the third (Q3) and fourth (Q4) quartile had a greater development of incidental NAFLD compared to the lowest quartile (Q1)[(OR = 2.86; 95 % CI: 1.20–6.81), (*P*-value = 0.017) and (OR = 5.97; 95 % CI: 2.54–14.01), (*P*-value < 0.001) for Q3 and Q4 compared to the Q1, respectively].

**Table 5:** The development of non- alcoholic fatty liver disease across quartiles of energy-adjusted daily calcium to magnesium ratio intake

	Energy-adjusted daily calcium to magnesium ratio intake						
	**Q1(*n* = 250)**	**Q2(*n* = 249)**	*P*-value^*^	**Q3(*n* = 250)**	*P*-value^*^	**Q4(*n* = 250)**	*P*-value^*^
Cases/control	27/223	34/215		49/201		86/164	
Range of Ca to Mg ratio	0.95 to 1.97	1.97 to 2.36		2.36 to 2.81		2.82 to 5.02	
^a^Model 1	1 (Ref)	1.30 (0.76–2.23)	0.33	2.01 (1.21–3.34)	**0.007**	4.33 (2.68–6.97)	**< 0.001**
^b^Model 2	1 (Ref)	1.22 (0.71–2.11)	0.46	1.93 (1.15–3.21)	**0.012**	4.45 (2.75–7.21)	**< 0.001**
^c^Model 3	1 (Ref)	1.57 (0.73–3.35)	0.24	2.89 (1.35–6.16)	**0.006**	5.72 (2.82–11.60)	**< 0.001**
^d^Model 4	1 (Ref)	1.82 (0.76–4.37)	0.17	2.86 (1.20–6.81)	**0.017**	5.97 (2.54–14.01)	**< 0.001**

Data are presented as odds ratio (95 %CI)

^*^Logistic regression

^a^crude model

^b^Adjusted for age and gender

^c^Additionally adjusted for body mass index, alcohol consumption, smoking, diabetes and physical activity

^d^Additionally adjusted for energy, dietary fiber, carbohydrate, fat, and protein intakes

The significance level: *P* < 0.05

To investigate the relationship between the energy-adjusted calcium to magnesium intake ratio and plasma levels of alanine aminotransferase (ALT) and aspartate aminotransferase (AST), the Pearson correlation test was conducted. Energy-adjusted calcium to magnesium intake ratio was positively correlated with plasma level of ALT (*r* = 0.18; *P* = 0.01); contrarily, it had no correlation with plasma levels of AST.

## Discussion

In this case control study, we observed that subjects with higher energy-adjusted dietary calcium to magnesium intake ratio had a greater development for incidental NAFLD, independent of confounding factors including age, gender, BMI, alcohol consumption, smoking, diabetes, physical activity, energy, dietary fiber, carbohydrate, fat, and protein intakes.

Recent studies have shown that the impact of nutritional factors on the incidence of NAFLD is more pronounced [32–35]. To the best of our knowledge, there is no study that has examined the relationship between the calcium to magnesium intake ratio and the development of NAFLD; however, calcium and magnesium were separately investigated as potential mediators in the pathogenesis of the disease. Based on consistent evidence, intake of magnesium was inversely associated to the factors related to the risk of insulin resistance [36], and metabolic syndrome, specifically factors such as high fasting glucose level, high waist circumference, and low high-density lipoprotein cholesterol [37, 38]. Lu et al. found that higher intakes of Mg during adulthood is related to a lower risk of NAFLD in middle age [22]. Moreover, in another study, subjects with NAFLD or alcoholic fatty liver were at higher risk of developing magnesium deficiency [39]. However, another study conducted in Canada was not able to find any links between magnesium intake and risk of NAFLD; considering the structural weaknesses in this study, the results might lack enough credibility to be deductible [40].

As mentioned beforehand in the present article, calcium has some biochemical interactions with magnesium. These interactions might have clinical manifestations concerning the pathogenesis of several metabolic disorders. Wenshuai Li et al. who investigated the data related to the Third National Health and Nutrition Examination Survey (NHANES III) follow-up US adults’ cohort [41], found out that the intake of magnesium was associated to an approximately 30 % reduced risk of NAFLD and prediabetes, only in subjects who consumed less than 1200 mg/day calcium. Their findings suggested that beneficial effect of magnesium might be attenuated when calcium intake is higher than the amount recommended by Dietary Reference Intakes (DRIs). One of the important mechanisms might be the suppressive effects of high calcium intake on the gastrointestinal absorption and renal reabsorption of magnesium which may alter the excretion of the ion in the feces and urine [24, 42].

As evident by the results of the present study, the Ca:Mg intake ratio might be a useful indicator to observe the combined impact of these two major minerals may exert upon different physiologic, as well as pathologic pathways. USDA food surveys from 1977 to 2007-8 show a rising food Ca:Mg ratio from 2.3 to 2.9 to 2.9–3.5 for all USA adults; these figures might be worthy of concern as they coincide with the rise of several metabolic diseases such as diabetes and colorectal cancer [43]. Furthermore, there is growing evidence indicating that modifications in serum Ca:Mg ratio is associated with some disorders including diabetes and metabolic syndrome [44], breast cancer [45, 46], cardiovascular diseases [47], and higher mortality [29]. Even though the optimal calcium to magnesium intake ratio is yet to be specified, some studies have suggested a 2:1 ratio as the optimum ratio [47]. Consistent with previous studies, our study indicated that participants in the third and fourth quartiles of Ca:Mg intake ratio (ranging from 2.36 to 5.02) had a significantly higher risk of NAFLD as compared to the first quartile.

The regulation of calcium and magnesium homeostasis in the body is interdependent. Calcium-sensitive receptor, also known as CaSR, is responsible to monitor plasma levels of both cations [48].Whenever the plasma level of each of the cations drops, CaSR up regulates the related mechanisms leading to an increase in its blood level, regardless of the other cation concentration in the blood [49, 50]. Furthermore, a drop in serum Mg could also decrease intracellular levels of Mg reducing the cellular Mg–ATP deposits. This may lead to an increase in Ca influx, which, in turn, may upgrade the Ca–ATP level of the cell. Increased intracellular calcium levels have been proposed an underlying mechanism for the pathogenesis of several metabolic and inflammatory disorders such as obesity, metabolic syndrome, diabetes, and NAFLD [45, 51–53].

This is the first observational study that evaluated the relationship between Ca:Mg intake ratio and the development of NAFLD. We were able to conduct the present research on a statistically acceptable sample size of subjects with corresponding socio-economic status. Moreover, the use of top-notch devices to determine the presence or the lack of the disease improved the accuracy and the precision of our inclusion/exclusion criteria. The present study also has some limitations. Case-control studies may prove an association but they do not demonstrate causation. Proving the causal relationship requires future intervention studies. Although we used a validated FFQ for measurement of dietary intakes, recall bias and measurement error are inevitable errors. Furthermore, although we tried to control the effect of major confounding factors, the effects of unknown factors and residual confounding cannot be ruled out. We also suggest that liver stiffness measurement (LSM) by Fibroscan be used in future studies for accurate prediction of liver fibrosis severity. Calculation of Fibrosis-4 (FIB-4) or NAFLD fibrosis score (NFS) which are indicators of advanced fibrosis in NAFLD patients used in exclusion of advanced fibrosis owing to its high negative predictive value is also recommended [54–56].

## Conclusions

The current study revealed that higher dietary calcium to magnesium intake ratio is associated with a greater development of NAFLD. Further interventional studies are needed to confirm the causal relationship between the dietary calcium to magnesium intake ratio and incidental NAFLD.

## Data Availability

The datasets used and/or analysed during the current study are available from the corresponding author on reasonable request.

## Abbreviations

NAFLD: Non-alcoholic fatty liver disease
CAP: Controlled Attenuation Parameter
FFQ: Food frequency questionnaire
MET: Metabolic equivalent
BMI: Body mass index
HDL-c: High density lipoprotein
FBS: Fasting blood sugar
TG: Triglycerides
LDL-c: Low-density lipoprotein
PA: Physical activity
AST: Aspartate aminotransferase
ALT: Alanine aminotransferase
CaSR: Calcium-sensitive receptor
